# A National Survey of Resources to Address Sepsis in Children in Tertiary Care Centers in Nigeria

**DOI:** 10.3389/fped.2019.00234

**Published:** 2019-06-11

**Authors:** Odiraa C. Nwankwor, Brianna McKelvie, Meg Frizzola, Krystal Hunter, Halima S. Kabara, Abiola Oduwole, Tagbo Oguonu, Niranjan Kissoon

**Affiliations:** ^1^Division of Critical Care Medicine, Department of Pediatrics, Alfred I. DuPont Hospital for Children, Wilmington, DE, United States; ^2^Division of Critical Care Medicine, Department of Pediatrics, Cooper University Hospital, Camden, NJ, United States; ^3^Department of Pediatrics, Children's Hospital, Western University, London, ON, Canada; ^4^Cooper University Hospital, Cooper Research Institute, Cooper Medical School of Rowan University, Camden, NJ, United States; ^5^Department of Anaesthesia/Intensive Care Unit, Aminu Kano Teaching Hospital, Kano, Nigeria; ^6^Department of Paediatrics, Lagos University Teaching Hospital/College of Medicine, University of Lagos, Lagos, Nigeria; ^7^Department of Paediatrics, University of Nigeria Teaching Hospital, Enugu, Nigeria; ^8^Department of Pediatrics and Emergency Medicine, University of British Columbia and BC Children's Hospital, Vancouver, BC, Canada

**Keywords:** sepsis, children, infections, guidelines, outcomes, Nigeria

## Abstract

**Background:** Infections leading to sepsis are major contributors to mortality and morbidity in children world-wide. Determining the capacity of pediatric hospitals in Nigeria to manage sepsis establishes an important baseline for quality-improvement interventions and resource allocations.

**Objectives:** To assess the availability and functionality of resources and manpower for early detection and prompt management of sepsis in children at tertiary pediatric centers in Nigeria.

**Methods:** This was an online survey of tertiary pediatric hospitals in Nigeria using a modified survey tool designed by the World Federation of Pediatric Intensive and Critical Care Societies (WFPICCS). The survey addressed all aspects of pediatric sepsis identification, management, barriers and readiness.

**Results:** While majority of the hospitals 97% (28/29) reported having adequate triage systems, only 60% (16/27) follow some form of guideline for sepsis management. There was no consensus national guideline for management of pediatric sepsis. Over 50% of the respondents identified deficit in parental education, poor access to healthcare services, failure to diagnose sepsis at referring institutions, lack of medical equipment and lack of a definitive protocol for managing pediatric sepsis, as significant barriers.

**Conclusions:** Certain sepsis-related interventions were reportedly widespread, however, there is no standardized sepsis protocol, and majority of the hospitals do not have pediatric intensive care units (PICU). These findings could guide quality improvement measures at institutional level, and healthcare policy/spending at the national level.

## Introduction

Infections leading to sepsis is a major cause of pediatric morbidity and mortality globally, however, 80% of infectious disease-related deaths occur in low and middle income countries (LMICs) (1). Children in LMICs are 18 times more likely to die under the age of five than children in higher income countries (HICs), a disparity caused by the interplay between economic, social and political factors and the inability to provide timely medical care partly due to resource limitations (2). The disparity in resources is starkly revealed by the findings of a continent wide survey showing that the Surviving Sepsis Campaign (SSC) guidelines can be fully implemented in only 1.5% (4/263) of African ICUs (3). Recognition of this resource gap led the “Global Intensive Care Working Group” of the European Society of Intensive Care Medicine to adapt the SSC guidelines to LIMCs (4–6).

In addition, defining sepsis could be challenging, and any definition needs to be context-appropriate for proper identification of patients. In resource rich environment, the criteria for defining sepsis syndrome include the presence or suspicion of infection associated with a systemic inflammatory response syndrome (SIRS). The SIRS is characterized by the presence of at least two of the four criteria below, one of which must be either an abnormal temperature or leucocyte count;

- Core temperature (rectal, bladder, or oral) of >101.3°F (>38.5°C) or <96.8°F (<36°C)
- Tachycardia more than 2 standard deviations (SD) above the normal or bradycardia (a mean heart rate <10th percentile for age) in children >1 year in the absence of external stimulus or drugs.
- Respiratory rate >2 SD above normal for age
- Leucocyte count elevated or depressed for the patient age

In LMICs, due to the limitations of SIRS, criteria for sepsis definition is based on clinical syndrome, clinical presentations and age of the patient. The suggested criteria for providers to make diagnosis of suspected sepsis in children include (7–9) proven or highly suspected infection and the presence of any two of the following;

- Abnormal temperature (axillary >38°C or <36°C) documented or by history^1^
- Abnormal heart rate for age: tachycardia or bradycardia (<1 year)^2^
- Respiratory insufficiency: tachypnea for age^3^
- Malaise and/or apathy^4^

See Table 1 for age-specific vital signs.

**Table 1:** Age-specific vital signs^a^.

**Age group**	**Tachycardia**	**Bradycardia**	**Respiratory rate**
0 days to 1 week	>180	<100	>50
1 week to 1 month	>180	<100	>40
1 month to 1 year	>180	<90	>34
2–6 years	>140	NA	>22
6–12 years	>130	NA	>18
13 to <18 years	>110	NA	>14

*NA, Not applicable*.

a *Modified from Goldstein et al.(9)*.

Assessing baseline prevalence of sepsis and septic shock in Nigeria is challenging due to dearth of epidemiological data on sepsis and septic shock in Nigeria and other LMICs. Literature reviews between 1980 and 2008 did not show any studies on the incidence, prevalence, mortality or case-fatality from sepsis in developing countries (10). However, the Sepsis Prevalence, Outcomes, and Therapy (SPROUT) Study found a point prevalence of severe sepsis of 23.1% across Africa, though only three sites from Africa were included in the study (11). More recently, a systemic review of the global burden of pediatric and neonatal sepsis found an aggregate estimate of 48 sepsis cases and 22 severe sepsis cases in children per 100,000 person-years globally but emphasized that due to lack of population-based data from low-resource settings, combined with lack of standardization of diagnostic criteria and sepsis definitions, it would be difficult to get an accurate estimation of global burden of pediatric sepsis (12).

Ultimately, Nigeria is the second highest contributor to under-five mortality (much due to sepsis) in the world (13). Determining the capacity of pediatric hospitals to manage sepsis establishes an important baseline from which to advocate for resources and for any quality-improvement interventions aimed at improving sepsis-related hospital outcomes. We therefore surveyed the heads of department of pediatrics at teaching hospitals and federal medical centers (tertiary pediatric centers) across the country to determine the resources available to manage children with sepsis. Our primary objective was to assess the availability and functionality of supplies and medications for the management of sepsis. Our secondary objectives included assessing the availability of emergency triage systems, skilled healthcare providers, use of guidelines and the barriers to early detection and prompt management of sepsis.

## Materials and Methods

This study was approved by the Institutional Review Board (IRB) of Nemours Office of Human Subject Protection/ Alfred I duPont Hospital for Children, Wilmington, Delaware USA. The institutions in Nigeria did not require IRB approval for the survey. The online survey instrument designed by the World Federation of Pediatric Intensive and Critical Care Societies (WFPICCS) was modified and used to address all aspects of pediatric sepsis identification, management, barriers, and readiness (14). We modified the survey to reflect the local health practices and environment in Nigeria, such as replacing the Pediatric Specialist designation with Consultant Pediatrician, and the types of health facility as Teaching Hospitals, Federal Medical Centers (FMCs) and Other (Specialist Hospitals). The hospitals are divided into teaching and non-teaching hospitals. Out of 31 hospitals, 26 are teaching hospitals, and 5 are non-teaching hospitals. All the Federal Medical Centers (five in number) are non-teaching hospitals. Of the 26 teaching hospitals, 21 are owned and operated by the federal government, while the remaining 5 are state owned and operated.

The contacts of the Heads of Department of Pediatrics of the teaching hospitals and FMCs were obtained from the secretariat of the Pediatric Association of Nigeria (PAN). However, PAN did not have a comprehensive list of all the heads of departments, so we were unable to obtain this information for all the health institutions especially the Federal Medical Centers and the state owned teaching hospitals.

### Data Collection

The survey was distributed by e-mail with a cover letter explaining the purpose of the survey and a link to the online survey. A portable document format (PDF) attachment of a sample of the survey was also available online to provide respondents an opportunity to review the questions and to obtain the data needed in answering the actual online survey. One survey per institution was sent to be completed by the Heads of Department of Pediatrics or their designee. While the survey did not contain any questions that would identify either the respondent or the institution, we included questions to identify the type of institution. The survey data was collected and managed using Survey Monkey tools housed at Cooper Medical School of Rowan University, Camden New Jersey, USA.

We assessed the availability and functionality of the medical supplies, equipment and medications at the healthcare institutions (see Supplementary Material for a copy of the survey tool).

### Statistical Analysis

We included all the returned surveys in our analysis. N value represented the total responses for a particular question in the survey. The data was summarized by providing a descriptive analysis where frequencies and percents were reported.

## Results

### Basic Characteristics of the Hospitals

Thirty-one surveys representing 31 of about 59 tertiary hospitals and Federal Medical Centers (FMCs) in Nigeria were completed and included in the analysis. Twenty-six (84%) of these were teaching hospitals while five (16%) were Federal Medical Centers. The federal teaching hospitals are well-established academic centers while some of the state-owned teaching hospitals and FMCs are either newly established or have smaller pediatric departments. The median age of children seen at these hospitals was 14 years (25.5 days-16.3 years), Over a period of 12 months, the respondents reported a total of 44,337 hospital visits, 12,912 emergency room visits, and an average of 881 patient were evaluated for sepsis in the emergency rooms, and 553 had a primary diagnosis of sepsis. They reported a median of 77 pediatric in-patient beds (range 30–190). All the surveys were completed by the Heads of Department of Pediatrics at these institutions.

### Triage and Emergency Care

**Figure 1:**
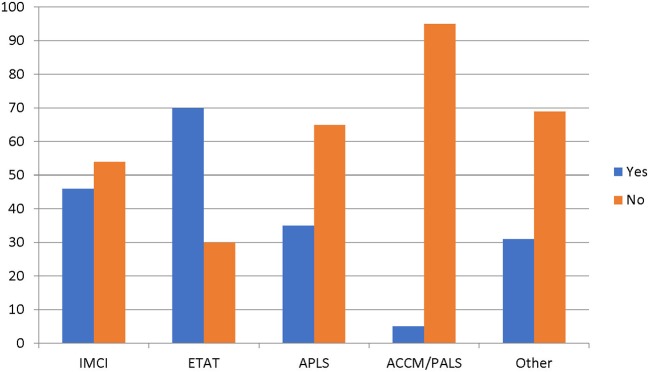
Specific guidelines used to prioritize sick children. IMCI, Integrated Management of Children Illnesses; ETAT, Emergency Triage, Assessment and Treatment; APLS, Advanced Pediatric Life Support; ACCM/PALS, American College of Critical Care Medicine/Pediatric Advanced Life Support.

All the hospitals reported having separate emergency rooms or acute care areas that were open 24 hours a day, and majority of them 97% (28/29) have systems in place to screen and prioritize very sick children. The resident doctors and nurse were primarily responsible for the triage process, and were trained on the use of The Emergency Triage Assessment and Treatment (ETAT) and the Integrated Management of Childhood Illnesses (IMCI) for screening and prioritizing severely ill children (15, 16). The Advanced Pediatric Life Support (APLS) or the American College of Critical Care Medicine/Pediatric Advanced Life Support (ACCM/PALS) guidelines were not commonly used (17, 18) (Figure 1). Children with suspected sepsis were reported to receive priority emergency attention. The median time reported from arrival to prioritization was 12.5 minutes, while the median time from prioritization to being seen by a physician was 20 minutes.

### Patient Care in the 1st Hour

Patients with suspected sepsis were reported to receive fluid boluses, antibiotics, and were evaluated by the Consultant Pediatrician within the first hour of presentation to the hospital (Table 2).

**Table 2:** First hour priority for a child with suspected sepsis in the ED or ward.

	**Yes % (N)**	**No % (N)**	**Total number of responses from 31 Hospitals**
Vascular access obtained	97 (28/29)	3 (1/29)	29
Crystalloid bolus given	74 (20/27)	26 (7/27)	27
Antibiotics given	96 (27/28)	4 (1/28)	28
Central line started	7 (2/27)	93 (25/27)	27
Arterial line started	4 (1/27)	96 (26/27)	27
Blood culture sent	79 (22/28)	21 (6/28)	28
Chest X-ray obtained	28 (7/25)	72 (18/25)	25
Blood gas obtained	7 (2/27)	93. (25/27)	27
Attending physician sees patient	89 (25/28)	11 (3/28)	28

Procedures or clinical decision made within the 1^st^ hour of presentation to the emergency room or pediatric ward with suspected sepsis

Total number of hospitals surveyed was 31

Number of hospitals that responded to a particular survey equals to N.

Intravenous Ceftriaxone and Gentamicin were the two commonly used antibiotics combination, and the resident doctors were reported to make this decision in 90% of cases. All the hospitals in this survey reported having resources to do complete blood count (CBC) and blood culture. Additionally, 86% (25/29) of the hospitals reported having the capacity to do blood smears for malaria parasites, while 83% (24/29) could do blood chemistry, and 72% (21/29) had resources to do urinalysis. However, they do not routinely do blood gases or coagulation profile. Fifty two percent of the hospitals reported that children who were severely ill in the emergency department were transferred to a designated special care area.

### In-patient Care

All acutely ill children with sepsis who were transferred to the pediatric wards were seen first by the resident doctors who would evaluate and institute therapy. The pediatric wards were open 24 h a day, seven days a week at most hospitals. On the average, there were seventy-three pediatric beds on the ward, and majority of the hospitals had beds that were designated for acutely ill children, however, there were no Pediatric Intensive Care Units (PICU) in 75%, (15/20) of the tertiary pediatric hospitals.

### Resources for Monitoring

Of all the hospitals that responded to this question, 100% (28/28) reported that they had pulse oximetry machine while 93% (25/27) had non-invasive blood pressure machines available at the hospitals, however, only 52% (13/25) had cardiac monitors. While 60% (16/27) of the hospitals stated that they follow some form of guidelines while treating sepsis, there was no consensus on any particular guideline. The guidelines listed ranged from departmental guideline, unit protocol and institutional guideline. The providers reportedly relied mainly on clinical examinations to guide sepsis management. Overall, 93% (26/28 and 25/27) used improvement in mental status and urine output, respectively, as their goal during sepsis management. Additionally, 88% (22/25), 85% (22/26), and 85% (23/27) of the respondents would use reduction in heart rate, improvement in blood pressure and improvement in peripheral perfusion, respectively as their goal directed targets in sepsis management. Only 13% (2/15) of institutions used serum markers such as lactate.

### Co-morbid Conditions

Protein-energy malnutrition was the commonest 92%, (24/26) co-morbid condition reported among children with sepsis in our study. Other co-morbid illnesses reported include malaria, Human Immunodeficiency Virus (HIV) infection, measles, sickle cell disease, anemia, malignancies and congenital heart diseases. Of these, malaria 100%, (25/25), Staphylococcus aureus 92%, (22/24), Streptococcus pneumonia 57%, (13/24) and Gram-negative rods 83%, (20/24) were reported as the most common causes of mortality among children who died from sepsis. Over a period of 1 year, respondents reported that an average of 15% (82/553) of children admitted with the diagnosis of sepsis died.

### Barriers to Sepsis Management

The common barriers to appropriate sepsis management in children reported were lack of parental awareness of early signs of sepsis 77%, (22/30), poor access to healthcare services 73%, (22/30), failure to diagnose sepsis at the referring health institution (67%, 20/30), lack of medical equipment 67%, (20/30), and lack of a definitive protocol for managing pediatric sepsis 50%, (15/30) (Table 3).

**Table 3:** Barriers to sepsis management.

	**Agree/strongly agree % (*N* = 30)**	**Neutral % (*N* = 30)**	**Disagree/strongly disagree % (*N* = 30)**
Deficit in parental education	77 (22)	3 (1)	20 (6)
Access to healthcare services	73 (22)	3 (1)	23 (7)
Lack of medical equipment resources	67 (20)	6 (2)	27 (8)
Lack of vascular access and crystalloid resuscitation	23 (7)	10 (3)	67 (20)
Lack of antibiotics	17 (5)	0 (0)	83 (25)
Failure to diagnose sepsis by referring institutions	67 (20)	6 (2)	27 (8)
Not enough trained hospital personnel	40 (12)	7 (2)	53 (16)
No defined protocol for managing pediatric sepsis	50 (15)	10 (3)	40 (12)
Long triage waiting time	27 (8)	26 (8)	47 (14)

List of barriers to sepsis management in children

Total number of hospitals surveyed was 31

Number of hospitals that responded was 30

Figures show percent and absolute number of respondents.

Triage waiting time, adequate number of trained personnel, vascular access, fluids for resuscitation and availability of antibiotics were not considered strong barriers to sepsis management. Other important barriers reported include financial constraints among families (poverty, high cost of medications, and the need for out of pocket payment), late or delayed presentation to the hospital, transportation system with inaccessible roads, lack of clean water, lack of rapid diagnostic tests and other laboratory resources, and cultural barriers (need for father's approval before the mother could bring child to the hospital).

### Equipment and Supplies

There was reported availability of respiratory supplies for simple oxygen supplementation and bag-valve mask ventilation in both the emergency rooms and pediatric wards for children with sepsis; however, 81% (21/26) of the hospitals surveyed reported that they did not have mechanical ventilators in the emergency rooms, while 88% (22/26) did not have ventilators on the pediatric wards (Table 4). Similarly, 58% (15/26) of the hospitals reportedly did not have cardiac monitors in the emergency rooms while 85% (22/26) did not have monitors on the pediatric ward. Along the same line, 88% (22/25) of the respondents reported that they did not have central line kit either in the emergency room or the pediatric ward 88% (21/24). Ceftriaxone, Gentamicin, and anti-malaria medication were available in most of the hospitals. Only 50% (13/26) of the hospitals had 24 h laboratory services for WBC count, while less than half of the hospitals could not do blood, urine or CSF cultures on a 24-h basis (Table 5).

**Table 4:** Equipment and supplies.

**Equipment**	**Emergency room % (N)**	**Pediatric ward % (N)**
Amby bags and masks	100 (25/25)	96 (24/25)
Suction machine	100 (26/26)	100 (26/26)
Laryngoscope	96 (25/26)	85 (22/26)
Endotracheal tubes of different sizes	77 (20/26)	62 (16/26)
Oxygen source	96 (25/26)	96 (24/25)
Mechanical ventilators	11 (3/26)	8 (2/25)
Portable Chest Xray machine	19 (5/26)	8 (2/25)
Blood gas machine	15 (4/26)	8 (2/25)
Pulse oximetry	92 (24/26)	81 (21/26)
IV administration set	100 (25/25)	100 (25/25)
IV pump	27 (7/26)	12 (3/26)
IV cannula (Angiocatheter)	88 (23/26)	88 (23/26)
Central line kit	8 (2/25)	13 (3/24)
Arterial line kit	12 (3/26)	8 (2/24)
Blood pressure machine	88 (23/26)	81 (21/26)
Cardiac monitors	38 (10/26)	12 (3/26)
Electrocardiogram (EKG machine)	27 (7/26)	12 (3/26)

List of available key equipment and supplies

Represented as percent and fractions of respondents

Shown as number present in the Emergency Rooms and Pediatric Wards.

**Table 5:** Medications and laboratory services.

**Medications**	**Emergency room % (N)**	**Pediatric ward % (N)**
Ceftriaxone	88 (22/25)	88 (21/24)
Gentamicin	92 (24/26)	88 (23/26)
Vancomycin	36 (9/25)	38 (9/24)
Artemisinin	96 (24/25)	92 (23/25)
Quinine Injection	77 (20/26)	73 (19/26)
Adrenaline (Epinephrine)	100 (26/26)	96 (25/26)
Hydrocortisone	100 (26/26)	96 (25/26)
**24 h laboratory services availability**		
WBC	50 (13/26)	50 (13/26)
Blood culture	38 (10/26)	62 (16/26)
Urine culture	38 (10/26)	62 (16/26)
CSF culture	50 (13/26)	50 (13/26)

List of commonly used medications and 24-hours availability of laboratory services

Represented as percent and fractions of respondents

Shown as number present in the Emergency Rooms and Pediatric Wards

## Discussion

This study provides a survey of available resources for managing sepsis in children at tertiary pediatric centers in Nigeria. We surveyed only teaching hospitals and federal medical centers in Nigeria. These hospitals provide the highest level of care for children with varied disease conditions, and the resources available to them for managing pediatric sepsis would be considered the highest in the country, and the standard of practice that other healthcare facilities in the country aspire to. Our data showed that all the hospitals have emergency room and triage areas reserved for children, and they have systems in place to screen and prioritize very sick children. In this survey, the presence of systems at these hospitals that consistently screens and prioritizes very sick children were considered adequate triage systems, although we were unable to ascertain if this was indeed the case by direct observation. The emergency rooms and acute care areas are open for service 24 h a day, 7 days a week. Most of the hospitals used ETAT to triage patients, and this is consistent with the practice in other resource limited settings (19–21).

Children with sepsis were reported to be triaged and evaluated by a physician in a median time of 12.5 and 20 min, respectively, and they reportedly received fluid boluses and antibiotics within the first hour. This practice follows some of the international pediatric guidelines (the Society for Critical Medicine Medicine/Pediatric Advanced Life Support SCCM/PALS guidelines and the Surviving Sepsis Campaign, SSC guideline) that recommend administration of 20 ml/kg of crystalloids over 5 min, and the administration of 60 ml/kg in the first hour. However, in resource-limited settings, more cautious fluid boluses have been recommended for febrile children with poor perfusion and those that meet the WHO shock criteria, while blood transfusion is reserved for children with severe anemia (22–24).

Children with sepsis were reported to receive the 1st dose of antibiotics within the 1st hour of presentation to the hospital in this survey. This practice is in agreement with the guidelines from resource-rich countries, which recommends antibiotics administration within the first hour of recognizing sepsis or septic shock in resource limited settings (5). In our setting, the choice of antibiotics was a challenge due to lack of epidemiological data, poor microbiological laboratory capacity and reduced availability of antibiotics. In Nigeria, ceftriaxone and gentamicin were the combined antibiotics of choice, though WHO guidelines recommend gentamicin and penicillin for hospitalized neonates and children in resource-limited settings (25). The reason for the choice of ceftriaxone over penicillin was not elucidated in this study

Our findings were consistent with other studies in Sub-Saharan Africa and other resource-limited settings, which showed widespread lack of diagnostic facilities and capacity for sepsis management, making international guidelines irrelevant for sepsis definitions, diagnosis and treatment (7, 26, 27). We found that only about fifty percent of the hospitals have laboratory services for 24 h for basic laboratory tests as full blood count, basic metabolic profile, blood, and urine cultures. This is a perennial issue in African countries, where the availability, accessibility, and affordability of blood culture is a common problem (28). Only 2% of the hospitals surveyed in Nigeria do have the capacity to measure any serum markers like serum lactate. This could potentially lead to missed opportunities for early detection of sepsis, and loss of an important guide to sepsis treatment (29, 30). Additionally, physical barriers including long distances to formal healthcare facilities, poor road networks and inadequate transport systems adversely affect access to health care and consequently, delay in care delivery. Studies have shown that both infant and childhood mortality rates increased as the distance to health facilities increased, whereas in a village, merely increasing the number of maternity clinics by one was associated with a decrease in infant mortality (31, 32). In Nigeria, there is an established significant association between physical barrier (including distance to healthcare facility and transport cost) to accessing health care and under-five mortality (33).

We found inadequate systems and structures to support the delivery of adequate care to children with severe sepsis. Seventy-five percent of hospitals surveyed do not have pediatric intensive care units, and most do not have the capacity to provide mechanical ventilation to support children with sepsis who are in respiratory failure. This is worrisome because studies from LMICs showed that large volumes of fluid during resuscitation could lead to pulmonary edema, and up to half of these children might need intubation and mechanical ventilation (34, 35). Safe delivery of invasive positive pressure ventilation and monitoring require the availability of functional Pediatric Intensive Care Unit (PICU).

Establishing PICUs in LMICs require assembling the necessary equipment/supplies, training of healthcare providers, and commitment from the hospital leadership (36, 37). In Nigeria, poor healthcare financing poses a threat to the provision of adequate medical equipment, supplies and medications that are essential for the delivery of critical care services for children. In 2015, the health expenditure to Gross Domestic Product (GDP) in Nigeria was 3.56%, despite the fact that in 2001, Nigeria signed the Abuja declaration where 53 African countries pledged to devote 15% of their national budget to health (38).

It was quite revealing in our study that almost all (92%) patients with sepsis were malnourished, and 15% of these patients died in the year preceding our study. This is consistent with other studies that showed high case fatality rate in children with septic shock and severe acute malnutrition, with mortality rate as high as 40% (39, 40). To date, issues related to fluid resuscitation in the malnourished child remain unsettled. Obonyo et al. reported in a prospective observational study that malnourished children showed no compromised cardiac function in response to fluid loading, and this could support the adoption of a more liberal approach to fluid resuscitation (41, 42). However, based on of constellation of evidence cautious approach and frequent re-evaluation is recommended (43). Acute malnutrition was defined here as children who were−2 Z-score in weight for height or bilateral pitting edema (44). The hospitals all kept data on the nutritional status of all children attending their institutions.

Another area in our study that negatively impacts sepsis management in children is the scanty data on the resistance pattern of the bacteria causing diseases in most Sub-Saharan African countries. The unregulated sale of antibiotics in shops and drug stores in most communities, the use in animal husbandry, and the widespread use in clinics and hospitals based on clinical syndromes rather than on laboratory sensitivity tests promote emergence of resistance (45, 46). In Nigeria, the key factors promoting antimicrobial resistance include unregulated antibiotics sale, proliferation of unlicensed medicine stores, shortage of licensed prescribers, poor antimicrobial resistance (AMR) awareness and use of antibiotics in animals without prescription (47).

Lack of parenteral awareness of sepsis and inability of referring healthcare providers to identify sepsis were among the significant barriers to sepsis management in over 50% of the respondents. Similarly, an international survey in Europe and United States showed that a mean of 88% of the interviewee have never heard the word “sepsis” (48). Even among healthcare workers, there was knowledge deficit with regards to sepsis awareness (49–51). In this study, the definitions of deficit in parental education and lack of parental awareness were discerned by the providers of care and was their judgement based on history taking.

Since information-seeking behaviors related to sepsis usually follow awareness campaigns (52), we recommend increased public awareness about sepsis. This could be in the form of public service announcements in print and electronic media (including radio and television), in major Nigerian languages. The Ministries of Health and Information could leverage the wide use of social media in Nigeria, especially WhatsApp to push information on sepsis The Medical and Dental Council of Nigeria (MDCN), the apex regulatory agency for medical practitioners in Nigeria, could require Sepsis Awareness CME credits for healthcare providers for their license renewals. Health policy makers in Nigeria should consider honoring the 2001 Abuja Declaration that pledged 15% of the National Budget to healthcare expenditures. Further studies could explore drafting of a national pediatric sepsis guideline, and assessing the acceptability and use of the new guideline.

### Limitations of our Study

We surveyed only tertiary pediatric centers in Nigeria, which might not reflect all the pediatric care in Nigeria, and we had no way to verify the responses that were self-reported. In addition, we did not obtain data to determine the outcomes at these institutions.

### Strength of our Study

This was a nation-wide survey with national and international collaborations. Our survey tool was a modification of a survey instrument designed by the World Federation of Pediatric Intensive and Critical Societies (WFPICCS). The findings and recommendations from this study could form a basis for the creation and adoption of Pediatric Sepsis Guideline in Nigeria.

## Conclusion

In our survey of tertiary care pediatric hospitals in Nigeria we found that certain sepsis-related interventions were widespread (e.g., adequate triage systems, administration of antibiotics within an hour) while others were neither available nor accessible at most hospitals (e.g., PICUs, availability of laboratory services 24 h/day, sepsis protocol). This information can be used to guide policy, healthcare spending and quality improvement interventions aimed at improving sepsis related outcomes in children.

## Data Availability

The datasets generated for this study are available on request to the corresponding author.

## Author Contributions

ON and BM wrote the initial manuscript and revisions for the final draft. MF, HK, AO, and TO contributed in interpreting the results, reviewing, and writing the manuscript. KH created the online survey tool, housed the database and provided data analysis. NK drafted the modified survey and had overall oversight of proof reading and editing the manuscript.

### Conflict of Interest Statement

The authors declare that the research was conducted in the absence of any commercial or financial relationships that could be construed as a potential conflict of interest.

## Footnotes

^1^Modified from Kortz et al. (8).

^2^Modified from Kortz et al. (8).

^3^Modified from Kortz et al. (8).

^4^Modified from Dunser et al. (7).
