# Supplementary material for: A National Survey of Resources to Address Sepsis in Children in Tertiary Care Centers in Nigeria
Source: Front Pediatr. 2019 Jun 11;7:234. doi: 10.3389/fped.2019.00234 (PMC6579914; doi:10.3389/fped.2019.00234)
Supplement: Supplementary file 1 [file Data_Sheet_1.PDF]

# Sepsis Survey

**PLEASE COMPLETE THE FOLLOWING SURVEY BY FILLING IN THE BLANKS PROVIDED. Some questions will require you to check the box associated with your response. FOR QUESTIONS, please contact either Dr. Nwankwor at odiraa.nwankwor@nemours.org or the Data Manager, Krystal Hunter at hunter-krystal@cooperhealth.edu.**

1. What is your title?

- ◯ Consultant Pediatrician
- ◯ Senior Registrar - Pediatrics
- ◯ Registrar - Pediatrics
- ◯ House Officer - Pediatrics
- ◯ Medical Officer
- ◯ Other (please specify)

____________________

2. Healthcare Facility affiliation

- ◯ Federal Government
- ◯ State Government

3. Type of healthcare facility

- ◯ Teaching Hospital
- ◯ Federal Medical Center
- ◯ Other (please specify)

____________________

Sepsis Survey

## Important barriers to appropriate sepsis therapy in your setting:

4. In the area where you practice, think about the most important barriers to appropriate sepsis therapy for children. Mark
how strongly you agree/disagree with the following as barriers to sepsis therapy.

| | Strongly Disagree | Disagree | Neutral | Agree | Strongly Agree |
|---|---|---|---|---|---|
| Deficits in parental education | ○ | ○ | ○ | ○ | ○ |
| Access to health care services | ○ | ○ | ○ | ○ | ○ |
| Lack of medical equipment resources | ○ | ○ | ○ | ○ | ○ |
| Lack of vascular access and crystalloid resuscitation | ○ | ○ | ○ | ○ | ○ |
| Lack of antibiotics | ○ | ○ | ○ | ○ | ○ |
| Failure to diagnose sepsis by referring facilities | ○ | ○ | ○ | ○ | ○ |
| Not enough trained hospital person | ○ | ○ | ○ | ○ | ○ |
| No defined protocol for managing pediatric sepsis | ○ | ○ | ○ | ○ | ○ |
| Long triage waiting times | ○ | ○ | ○ | ○ | ○ |

5. What other barriers exist for delivering sepsis therapy to children? List them below

## ACUTE CARE/EMERGENCY MANAGEMENT AREA (FIRST CONTACT WITH HOSPITAL)

6. Is there an acute care area or emergency management area where acutely ill/injured children are managed?

- ○ Yes
- ○ No

7. Is the paediatric acute care area separate from the adult area?

- ○ Yes
- ○ No

8. How many hours/day is the pediatric acute care area functional?

[ ]

9. Is the acute care area open on weekends?

- ○ Yes
- ○ No

10. Is there a system to screen and prioritize very sick children?

- ○ Yes
- ○ No

11. Who is responsible for prioritizing?

- ☐ Pediatric Specialists/Consultants
- ☐ Pediatric Residents
- ☐ General Physicians
- ☐ Medical Officers
- ☐ Interns
- ☐ Nurses
- ☐ Nursing Aides
- ☐ Other

12. What is the average time from arrival to prioritization?*(Please list time and units)*

13. What is the average time (units below) from prioritization to being seen by a physician:*(Please list time and units)*

14. Are any specific guidelines used for prioritization:

| | Yes | No |
|---|---|---|
| IMCI (Integrated Management of Childhood Illnesses) | ◯ | ◯ |
| ETAT (Emergency Triage Assessment and Treatment) | ◯ | ◯ |
| APLS (Advanced Pediatric Life Support) | ◯ | ◯ |
| ACCM/PALS (American College of Critical Care Medicine/Pediatric Advanced Life Support) | ◯ | ◯ |
| Other | ◯ | ◯ |

15. If you stated "Other" in the above question, can you specify the other guidelines?

* 16. Are children with suspected sepsis given emergency priority?

◯ Yes

◯ No

17. Describe how these children are given priority.

* 18. Are the staff trained in screening and prioritization of the severely ill children?

- Yes
- No

19. What specific training do they received?

| | Yes | No |
|---|---|---|
| IMCI (Integrated Management of Childhood Illnesses | ◯ | ◯ |
| ETAT (Emergency Triage Assessment and Treatment) | ◯ | ◯ |
| APLS (Advanced Pediatric Life Support) | ◯ | ◯ |
| ACCM/PALS (American College of Critical Care Medicine/Pediatric Advanced Life Support) | ◯ | ◯ |
| Other training | ◯ | ◯ |

20. If you stated that you received other training, can you specify?

## PEDIATRIC WARD/INPATIENT AREA (HOSPITALIZED CHILDREN)

21. How many total pediatric beds are in the facility?

* 22. Are there specific beds designated for acutely ill children on the unit/ward?

- Yes
- No

23. How many?

[ ]

24. Are these beds located on the ward?

- ◯ Yes
- ◯ No

25. Are these beds located in a designated pediatric ICU?

- ◯ Yes
- ◯ No

26. Do you have an infectious disease (isolation) ward?

- ◯ Yes
- ◯ No

27. Do patients come with referral notes when they have been referred from first level units?

- ◯ Never
- ◯ Sometimes
- ◯ Often
- ◯ Most of the time
- ◯ Always

28. Comments:

[ ]

## What is the distribution of ward staff ?

29. Is there a physician on call 24 hours?

- ◯ Yes
- ◯ No

30. Who is the frontline care provider?

- ☐ Pediatric Specialists/Consultants
- ☐ Pediatric residents
- ☐ General physicians
- ☐ Interns
- ☐ Medical Officer
- ☐ Other (please specify)

31. Who receives the new patients on the ward?

- ☐ Pediatric Specialists/Consultants
- ☐ Pediatric Residents
- ☐ General Physicians
- ☐ Medical Officers
- ☐ Interns
- ☐ Nurses
- ☐ Nursing Aides
- ☐ Other (please specify)

32. DESCRIBE what would happen to a child with suspected SEPSIS sent to ER and the ward/ICU **within an hour**:

| | Yes | No |
|---|---|---|
| Vascular access obtained | ◯ | ◯ |
| Crystalloid bolus given | ◯ | ◯ |
| Antibiotics given | ◯ | ◯ |
| Central Line started | ◯ | ◯ |
| Arterial line started | ◯ | ◯ |
| Blood cultures sent | ◯ | ◯ |
| Chest X-Ray obtained | ◯ | ◯ |
| Blood gas obtained | ◯ | ◯ |
| Attending physician sees patient | ◯ | ◯ |

33. Who sees the patient first?

- [ ] Pediatric Specialists/Consultants
- [ ] Pediatric Residents
- [ ] General Physicians
- [ ] Medical Officers
- [ ] Interns
- [ ] Nurses
- [ ] Nursing Aides
- [ ] Other (please specify)

34. Who reviews the patient?

- [ ] Pediatric specialists
- [ ] Pediatric residents
- [ ] General physicians
- [ ] Medical Officers
- [ ] Interns
- [ ] Nurses
- [ ] Nursing Aides
- [ ] Other (please specify)

35. How is the child's severity of illness determined?

- [ ] Physical exam alone
- [ ] Algorithm (IMCI or ETAT)
- [ ] Institutional protocol
- [ ] Scoring system
- [ ] Lab values
- [ ] Other (please specify)

36. Who is responsible for achieving vascular access?

- [ ] Pediatric Specialists/Consultants
- [ ] Pediatric Residents
- [ ] General Physicians
- [ ] Medical Officers
- [ ] Interns
- [ ] Nurses
- [ ] Nursing Aides
- [ ] Other (please specify)

37. Who is responsible for administering crystalloid boluses?

- [ ] Pediatric Specialists/Consultants
- [ ] Pediatric Residents
- [ ] General Physicians
- [ ] Medical Officers
- [ ] Interns
- [ ] Nurses
- [ ] Nursing Aides
- [ ] Other (please specify)

38. What PERCENTAGE of patients with sepsis receive IV fluid resuscitation according to the PALS/ACCM guidelines? [up
to and over 60ml/kg in the first 15 minutes titrated to improved perfusion and evaluating for development of rales/hepatomegaly]

39. Who prescribes antibiotics?

- [ ] Pediatric specialists/Consultants
- [ ] Pediatric Residents
- [ ] General Physicians
- [ ] Interns
- [ ] Medical Officer
- [ ] Other (please specify)

40. Which antibiotics are most commonly used for sepsis?

- [ ] Ampicillin
- [ ] Penicillin
- [ ] Vancomycin
- [ ] Ceftriaxone
- [ ] Gentamicin
- [ ] Chloramphenicol
- [ ] Other: Quinine, Coartemisin

41. What percentage of pediatric patients with sepsis receives antibiotics:

Within 1 hour

Within 2 hours

42. What labs are usually ordered for a patient with sepsis?

- [ ] Complete blood count
- [ ] Chemistry
- [ ] Blood gas
- [ ] Coagulation profile
- [ ] Urinalysis
- [ ] Blood smear for malaria
- [ ] Other
- [ ] Other additional
- [ ] Specify Other lab test and additional other lab tests

43. Blood cultures

What percentage of patients have a blood culture obtained

What percentage of blood cultures yield an isolate that is helpful for patient management?

* 44. If a patient becomes severely ill, are they transferred to another area?

- ◯ Yes
- ◯ No

45. Where are they transferred?

- [ ] Intensive care unit
- [ ] Another hospital
- [ ] Other (please specify)

46. What equipment is available for patient monitoring?

| | Yes | No | | | |
|---|---|---|---|---|---|
| Cardiac monitor | ○ | ○ | ○ | ○ | ○ |
| Pulse oximeter | ○ | ○ | ○ | ○ | ○ |
| Blood pressure machine | ○ | ○ | ○ | ○ | ○ |
| Other | ○ | ○ | ○ | ○ | ○ |

Specify other equipment

47. Are there any specific guidelines used when treating a child with sepsis?

○ Yes

○ No

48. Which guidelines are used?

49. What goals are used to direct sepsis therapy?

| | Yes | No |
|---|---|---|
| Reduction in Heart rate | ◯ | ◯ |
| Improvement in blood pressure | ◯ | ◯ |
| Improvement in mental status | ◯ | ◯ |
| Urine Output | ◯ | ◯ |
| Improved peripheral perfusion | ◯ | ◯ |
| Serum markers (lactate, SVO2) | ◯ | ◯ |

Other Goals:

## SEPSIS STATISTICS IN YOUR INSTITUTION

50. What types of patients are seen in this area? (please list ages, diagnoses, etc)

Youngest Age (In years) [ ]

Oldest Age (In Years) [ ]

51. What events do you serve?

| | Yes | No |
|---|---|---|
| Medical emergencies | ○ | ○ |
| Trauma | ○ | ○ |
| Surgical emergencies | ○ | ○ |

52. What are the common comorbidities that exist in patients with sepsis?

| | Yes | No |
|---|---|---|
| Tuberculosis | ○ | ○ |
| Malnutrition | ○ | ○ |
| Other | ○ | ○ |

Specify other comorbidity:

[ ]

53. What organism(s) is/are responsible for most childhood deaths due to sepsis - BACTERIAL?

- [ ] Streptococcus pneumoniae
- [ ] Group A Strep
- [ ] Staphylococcus aureus
- [ ] Gram negative rods
- [ ] Other
- [ ] Specify "other" bacteria:

[ ]

54. What organism(s) is/are responsible for most childhood deaths due to sepsis - VIRAL? (Please list)

[ ]

55. What organism(s) is/are responsible for most childhood deaths due to sepsis - PROTOZOAL?

☐ Malaria

☐ Other

☐ Specify "other" Protozoa:

____________________

56. What organism(s) is/are responsible for most childhood deaths due to sepsis - FUNGAL? (Please list)

____________________

57. Please define how many time the below happened in the previous calendar year (January to December 2016)?

| | |
|---|---|
| Patient visits | |
| Patient visits to the emergency department | |
| Patients evaluated for sepsis in the emergency department | |
| Inpatient Admissions | |
| Inpatient admissions with a primary diagnosis of sepsis | |
| Inpatient Deaths | |

## RESUSCITATION FACILITIES: DRUGS AND OTHER SUPPLIES

**Indicate if the following items are available in the Acute Care/ Emergency Area and in the Pediatric ward. Provide any additional remarks.**

58. Job aides/guidelines for management of childhood emergencies:

| | Available | Not Available | Available but not functional |
|---|---|---|---|
| Acute care/ Emergency Area | ◯ | ◯ | ◯ |
| Pediatric ward | ◯ | ◯ | ◯ |

Remarks

59. EQUIPMENT & SUPPLIES: Resuscitation Table

| | Available | Not Available | Available but not functional |
|---|---|---|---|
| Acute care/ Emergency Area | ◯ | ◯ | ◯ |
| Pediatric ward | ◯ | ◯ | ◯ |

Remarks

60. EQUIPMENT & SUPPLIES: Suction machines

| | Available | Not Available | Available but not functional |
|---|---|---|---|
| Acute care/ Emergency Area | ◯ | ◯ | ◯ |
| Pediatric ward | ◯ | ◯ | ◯ |

Remarks

61. EQUIPMENT & SUPPLIES: Ambu bag and masks

| | Available | Not Available | Available but not functional |
|---|---|---|---|
| Acute care/ Emergency Area | ○ | ○ | ○ |
| Pediatric ward | ○ | ○ | ○ |

Remarks

62. EQUIPMENT & SUPPLIES: Oxygen source

| | Available | Not Available | Available but not functional |
|---|---|---|---|
| Acute care/ Emergency Area | ○ | ○ | ○ |
| Pediatric ward | ○ | ○ | ○ |

Remarks

63. EQUIPMENT & SUPPLIES: Nasal prongs/cannula/catheter

| | Available | Not Available | Available but not functional |
|---|---|---|---|
| Acute care/ Emergency Area | ○ | ○ | ○ |
| Pediatric ward | ○ | ○ | ○ |

Remarks

64. EQUIPMENT & SUPPLIES: Facemask (simple or non-rebreather)

| | Available | Not Available | Available but not functional |
|---|---|---|---|
| Acute care/ Emergency Area | ○ | ○ | ○ |
| Pediatric ward | ○ | ○ | ○ |

Remarks

65. EQUIPMENT & SUPPLIES: IV administration sets

| | Available | Not Available | Available but not functional |
|---|---|---|---|
| Acute care/ Emergency Area | ○ | ○ | ○ |
| Pediatric ward | ○ | ○ | ○ |

Remarks

66. EQUIPMENT & SUPPLIES: Infusion pumps

| | Available | Not Available | Available but not functional |
|---|---|---|---|
| Acute care/ Emergency Area | ○ | ○ | ○ |
| Pediatric ward | ○ | ○ | ○ |

Remarks

67. EQUIPMENT & SUPPLIES: Needle system for IV infusion (i.e. butterflies or scalp vein sets)

| | Available | Not Available | Available but not functional |
|---|---|---|---|
| Acute care/ Emergency Area | ○ | ○ | ○ |
| Pediatric ward | ○ | ○ | ○ |

List Sizes:

68. EQUIPMENT & SUPPLIES: Intravenous cannulae (angiocaths, jelcos, venflons)

| | Available | Not Available | Available but not functional |
|---|---|---|---|
| Acute care/ Emergency Area | ○ | ○ | ○ |
| Pediatric ward | ○ | ○ | ○ |

Remarks

69. EQUIPMENT & SUPPLIES: Central line kits

| | Available | Not Available | Available but not functional |
|---|---|---|---|
| Acute care/ Emergency Area | ◯ | ◯ | ◯ |
| Pediatric ward | ◯ | ◯ | ◯ |

Remarks

70. EQUIPMENT & SUPPLIES: Arterial line kits

| | Available | Not Available | Available but not functional |
|---|---|---|---|
| Acute care/ Emergency Area | ◯ | ◯ | ◯ |
| Pediatric ward | ◯ | ◯ | ◯ |

Remarks

71. EQUIPMENT & SUPPLIES: Blood giving sets

| | Available | Not Available | Available but not functional |
|---|---|---|---|
| Acute care/ Emergency Area | ◯ | ◯ | ◯ |
| Pediatric ward | ◯ | ◯ | ◯ |

Remarks

72. EQUIPMENT & SUPPLIES: Torch

| | Available | Not Available | Available but not functional |
|---|---|---|---|
| Acute care/ Emergency Area | ◯ | ◯ | ◯ |
| Pediatric ward | ◯ | ◯ | ◯ |

Remarks

73. EQUIPMENT & SUPPLIES: Oropharyngeal airways

| | Available | Not Available | Available but not functional |
|---|---|---|---|
| Acute care/ Emergency Area | ○ | ○ | ○ |
| Pediatric ward | ○ | ○ | ○ |

Remarks

74. EQUIPMENT & SUPPLIES: Oropharyngeal airways

| | Available | Not Available | Available but not functional |
|---|---|---|---|
| Acute care/ Emergency Area | ○ | ○ | ○ |
| Pediatric ward | ○ | ○ | ○ |

Remarks

75. EQUIPMENT & SUPPLIES: Laryngoscope

| | Available | Not Available | Available but not functional |
|---|---|---|---|
| Acute care/ Emergency Area | ○ | ○ | ○ |
| Pediatric ward | ○ | ○ | ○ |

Remarks

76. EQUIPMENT & SUPPLIES: Endotracheal tubes (all sizes)

| | Available | Not Available | Available but not functional |
|---|---|---|---|
| Acute care/ Emergency Area | ○ | ○ | ○ |
| Pediatric ward | ○ | ○ | ○ |

Remarks

77. EQUIPMENT & SUPPLIES: Mechanical ventilator

| | Available | Not Available | Available but not functional |
|---|---|---|---|
| Acute care/ Emergency Area | ○ | ○ | ○ |
| Pediatric ward | ○ | ○ | ○ |

Remarks

78. EQUIPMENT & SUPPLIES: Source of Heat

| | Available | Not Available | Available but not functional |
|---|---|---|---|
| Acute care/ Emergency Area | ○ | ○ | ○ |
| Pediatric ward | ○ | ○ | ○ |

Remarks

79. EQUIPMENT & SUPPLIES: Thermometer

| | Available | Not Available | Available but not functional |
|---|---|---|---|
| Acute care/ Emergency Area | ○ | ○ | ○ |
| Pediatric ward | ○ | ○ | ○ |

Remarks

80. EQUIPMENT & SUPPLIES: Naso-gastric tubes

| | Available | Not Available | Available but not functional |
|---|---|---|---|
| Acute care/ Emergency Area | ○ | ○ | ○ |
| Pediatric ward | ○ | ○ | ○ |

Remarks

81. EQUIPMENT & SUPPLIES: Antiseptic (for cleaning the skin)

| | Available | Not Available | Available but not functional |
|---|---|---|---|
| Acute care/ Emergency Area | ○ | ○ | ○ |
| Pediatric ward | ○ | ○ | ○ |

Remarks

82. EQUIPMENT & SUPPLIES: Specimen bottles for microbiology

| | Available | Not Available | Available but not functional |
|---|---|---|---|
| Acute care/ Emergency Area | ○ | ○ | ○ |
| Pediatric ward | ○ | ○ | ○ |

Remarks

83. EQUIPMENT & SUPPLIES: Weighing machines

| | Available | Not Available | Available but not functional |
|---|---|---|---|
| Acute care/ Emergency Area | ○ | ○ | ○ |
| Pediatric ward | ○ | ○ | ○ |

Remarks

84. EQUIPMENT & SUPPLIES: Dextrose stix and glucometer

| | Available | Not Available | Available but not functional |
|---|---|---|---|
| Acute care/ Emergency Area | ○ | ○ | ○ |
| Pediatric ward | ○ | ○ | ○ |

Remarks

85. EQUIPMENT & SUPPLIES: Blood slides

| | Available | Not Available | Available but not functional |
|---|---|---|---|
| Acute care/ Emergency Area | ○ | ○ | ○ |
| Pediatric ward | ○ | ○ | ○ |

Remarks

86. EQUIPMENT & SUPPLIES: LP needles

| | Available | Not Available | Available but not functional |
|---|---|---|---|
| Acute care/ Emergency Area | ○ | ○ | ○ |
| Pediatric ward | ○ | ○ | ○ |

Remarks

87. EQUIPMENT & SUPPLIES: Blood gas machine

| | Available | Not Available | Available but not functional |
|---|---|---|---|
| Acute care/ Emergency Area | ○ | ○ | ○ |
| Pediatric ward | ○ | ○ | ○ |

Remarks

88. EQUIPMENT & SUPPLIES: Pulse oximeter

| | Available | Not Available | Available but not functional |
|---|---|---|---|
| Acute care/ Emergency Area | ○ | ○ | ○ |
| Pediatric ward | ○ | ○ | ○ |

Remarks

89. EQUIPMENT & SUPPLIES: Blood Pressure Machine (Pedi cuff)

| | Available | Not Available | Available but not functional |
|---|---|---|---|
| Acute care/ Emergency Area | ○ | ○ | ○ |
| Pediatric ward | ○ | ○ | ○ |

Remarks

90. EQUIPMENT & SUPPLIES: Cardiac monitoring (heart rate, EKG)

| | Available | Not Available | Available but not functional |
|---|---|---|---|
| Acute care/ Emergency Area | ○ | ○ | ○ |
| Pediatric ward | ○ | ○ | ○ |

Remarks

91. EQUIPMENT & SUPPLIES: EKG machine

| | Available | Not Available | Available but not functional |
|---|---|---|---|
| Acute care/ Emergency Area | ○ | ○ | ○ |
| Pediatric ward | ○ | ○ | ○ |

Remarks

92. EQUIPMENT & SUPPLIES: Portable Chest radiograph

| | Available | Not Available | Available but not functional |
|---|---|---|---|
| Acute care/ Emergency Area | ○ | ○ | ○ |
| Pediatric ward | ○ | ○ | ○ |

Remarks

93. EQUIPMENT & SUPPLIES: Clean water supply

| | Available | Not Available | Available but not functional |
|---|---|---|---|
| Acute care/ Emergency Area | ○ | ○ | ○ |
| Pediatric ward | ○ | ○ | ○ |

Remarks

94. DRUGS: Diazepam

| | Available | Not Available | Available but not functional |
|---|---|---|---|
| Acute care/ Emergency Area | ○ | ○ | ○ |
| Pediatric ward | ○ | ○ | ○ |

Remarks

95. DRUGS: Glucose 5%

| | Available | Not Available | Available but not functional |
|---|---|---|---|
| Acute care/ Emergency Area | ○ | ○ | ○ |
| Pediatric ward | ○ | ○ | ○ |

Remarks

96. DRUGS: Glucose 25%

| | Available | Not Available | Available but not functional |
|---|---|---|---|
| Acute care/ Emergency Area | ○ | ○ | ○ |
| Pediatric ward | ○ | ○ | ○ |

Remarks

97. DRUGS: Glucose 10%

| | Available | Not Available | Available but not functional |
|---|---|---|---|
| Acute care/ Emergency Area | ○ | ○ | ○ |
| Pediatric ward | ○ | ○ | ○ |

Remarks

98. DRUGS: Oral rehydration solution

| | Available | Not Available | Available but not functional |
|---|---|---|---|
| Acute care/ Emergency Area | ○ | ○ | ○ |
| Pediatric ward | ○ | ○ | ○ |

Remarks

99. DRUGS: Benzyl Penicillin

| | Available | Not Available | Available but not functional |
|---|---|---|---|
| Acute care/ Emergency Area | ○ | ○ | ○ |
| Pediatric ward | ○ | ○ | ○ |

Remarks

100. DRUGS: Vancomycin

| | Available | Not Available | Available but not functional |
|---|---|---|---|
| Acute care/ Emergency Area | ○ | ○ | ○ |
| Pediatric ward | ○ | ○ | ○ |

Remarks

101. DRUGS: Gentamicin

| | Available | Not Available | Available but not functional |
|---|---|---|---|
| Acute care/ Emergency Area | ○ | ○ | ○ |
| Pediatric ward | ○ | ○ | ○ |

Remarks

102. DRUGS: Ceftriaxone

| | Available | Not Available | Available but not functional |
|---|---|---|---|
| Acute care/ Emergency Area | ○ | ○ | ○ |
| Pediatric ward | ○ | ○ | ○ |

Remarks

103. DRUGS: Chloramphenicol injection

| | Available | Not Available | Available but not functional |
|---|---|---|---|
| Acute care/ Emergency Area | ○ | ○ | ○ |
| Pediatric ward | ○ | ○ | ○ |

Remarks

104. DRUGS: Quinine injection

| | Available | Not Available | Available but not functional |
|---|---|---|---|
| Acute care/ Emergency Area | ○ | ○ | ○ |
| Pediatric ward | ○ | ○ | ○ |

Remarks

105. DRUGS: Adrenaline (epinephrine)

| | Available | Not Available | Available but not functional |
|---|---|---|---|
| Acute care/ Emergency Area | ◯ | ◯ | ◯ |
| Pediatric ward | ◯ | ◯ | ◯ |

List concentration

106. DRUGS: Hydrocortisone

| | Available | Not Available | Available but not functional |
|---|---|---|---|
| Acute care/ Emergency Area | ◯ | ◯ | ◯ |
| Pediatric ward | ◯ | ◯ | ◯ |

Remarks

107. DRUGS: Normal Saline

| | Available | Not Available | Available but not functional |
|---|---|---|---|
| Acute care/ Emergency Area | ◯ | ◯ | ◯ |
| Pediatric ward | ◯ | ◯ | ◯ |

Remarks

108. DRUGS: Hartmann's Solution

| | Available | Not Available | Available but not functional |
|---|---|---|---|
| Acute care/ Emergency Area | ◯ | ◯ | ◯ |
| Pediatric ward | ◯ | ◯ | ◯ |

Remarks

109. DRUGS: Artemisinin

| | Available | Not Available | Available but not functional |
|---|---|---|---|
| Acute care/ Emergency Area | ○ | ○ | ○ |
| Pediatric ward | ○ | ○ | ○ |

Remarks

110. DRUGS: Tab Quinine

| | Available | Not Available | Available but not functional |
|---|---|---|---|
| Acute care/ Emergency Area | ○ | ○ | ○ |
| Pediatric ward | ○ | ○ | ○ |

Remarks

111. DRUGS: Cotrimoxazole

| | Available | Not Available | Available but not functional |
|---|---|---|---|
| Acute care/ Emergency Area | ○ | ○ | ○ |
| Pediatric ward | ○ | ○ | ○ |

Remarks

112. DRUGS: Furosemide

| | Available | Not Available | Available but not functional |
|---|---|---|---|
| Acute care/ Emergency Area | ○ | ○ | ○ |
| Pediatric ward | ○ | ○ | ○ |

Remarks

113. DRUGS: Calcium gluconate

| | Available | Not Available | Available but not functional |
|---|---|---|---|
| Acute care/ Emergency Area | ◯ | ◯ | ◯ |
| Pediatric ward | ◯ | ◯ | ◯ |

Remarks

114. DRUGS: Sodium bicarbonate

| | Available | Not Available | Available but not functional |
|---|---|---|---|
| Acute care/ Emergency Area | ◯ | ◯ | ◯ |
| Pediatric ward | ◯ | ◯ | ◯ |

Remarks

115. DRUGS: Other Drug #1

| | Available | Not Available | Available but not functional |
|---|---|---|---|
| Acute care/ Emergency Area | ◯ | ◯ | ◯ |
| Pediatric ward | ◯ | ◯ | ◯ |

Specify other drug

116. DRUGS: Other Drug # 2

| | Available | Not Available | Available but not functional |
|---|---|---|---|
| Acute care/ Emergency Area | ◯ | ◯ | ◯ |
| Pediatric ward | ◯ | ◯ | ◯ |

Specify other drug

117. DRUGS: Other Drug # 3

| | Available | Not Available | Available but not functional |
|---|---|---|---|
| Acute care/ Emergency Area | ○ | ○ | ○ |
| Pediatric ward | ○ | ○ | ○ |

Specify other drug

118. DRUGS: Other Drug # 4

| | Available | Not Available | Available but not functional |
|---|---|---|---|
| Acute care/ Emergency Area | ○ | ○ | ○ |
| Pediatric ward | ○ | ○ | ○ |

Specify other drug

## ASSESSMENT OF LABORATORY RESOURCES

119. Is the laboratory 24 hours?

- Yes
- No

120. What hours is it open?

Time Labs Opens (In 24hr)

Time Labs Closes (In 24hr)

121. Which of he following tests are done in the laboratory?

| | Yes - Available 24 hr | Yes, but not available 24 hr | No |
|---|---|---|---|
| Blood sugar | | | |
| Blood slide | | | |
| Blood film | | | |
| Hemoglobin/ PCV | | | |
| WBC count | | | |
| CSF microscopy | | | |
| Urine microscopy | | | |
| Blood grouping and cross match | | | |
| Blood culture | | | |
| Urine culture | | | |
| CSF culture | | | |
| Other | | | |

Other Lab Test Specify
